# Feather mites play a role in cleaning host feathers: New insights from DNA metabarcoding and microscopy

**DOI:** 10.1111/mec.14581

**Published:** 2018-05-03

**Authors:** Jorge Doña, Heather Proctor, David Serrano, Kevin P. Johnson, Arnika Oddy‐van Oploo, Jose C. Huguet‐Tapia, Marina S. Ascunce, Roger Jovani

**Affiliations:** ^1^ Department of Evolutionary Ecology Estación Biológica de Doñana (EBD‐CSIC) Sevilla Spain; ^2^ Department of Biological Sciences University of Alberta Edmonton AB Canada; ^3^ Department of Conservation Biology Estación Biológica de Doñana (EBD‐CSIC) Sevilla Spain; ^4^ Illinois Natural History Survey Prairie Research Institute University of Illinois at Urbana‐Champaign Champaign Illinois; ^5^ Department of Plant Pathology University of Florida Gainesville Florida; ^6^ Emerging Pathogens Institute University of Florida Gainesville Florida

**Keywords:** bacteria, birds, diet, DNA metabarcoding, ecological interactions, feather mites, fungi, high‐throughput sequencing, interactions, symbionts

## Abstract

Parasites and other symbionts are crucial components of ecosystems, regulating host populations and supporting food webs. However, most symbiont systems, especially those involving commensals and mutualists, are relatively poorly understood. In this study, we have investigated the nature of the symbiotic relationship between birds and their most abundant and diverse ectosymbionts: the vane‐dwelling feather mites. For this purpose, we studied the diet of feather mites using two complementary methods. First, we used light microscopy to examine the gut contents of 1,300 individual feather mites representing 100 mite genera (18 families) from 190 bird species belonging to 72 families and 19 orders. Second, we used high‐throughput sequencing (HTS) and DNA metabarcoding to determine gut contents from 1,833 individual mites of 18 species inhabiting 18 bird species. Results showed fungi and potentially bacteria as the main food resources for feather mites (apart from potential bird uropygial gland oil). Diatoms and plant matter appeared as rare food resources for feather mites. Importantly, we did not find any evidence of feather mites feeding upon bird resources (e.g., blood, skin) other than potentially uropygial gland oil. In addition, we found a high prevalence of both keratinophilic and pathogenic fungal taxa in the feather mite species examined. Altogether, our results shed light on the long‐standing question of the nature of the relationship between birds and their vane‐dwelling feather mites, supporting previous evidence for a commensalistic–mutualistic role of feather mites, which are revealed as likely fungivore–microbivore–detritivore symbionts of bird feathers.

## 1 INTRODUCTION

Symbionts (i.e., parasites, mutualists and commensalists that intimately interact with their hosts; Leung & Poulin, 2008) comprise the most diverse group of organisms on Earth (Dobson, Lafferty, Kuris, Hechinger, & Jetz, 2008; Larsen, Miller, Rhodes, & Wiens, 2017; Poulin & Morand, 2000, 2004). Symbionts are crucial for ecosystem stability: they regulate host populations and support food webs, where parasites alone are responsible for 75% of the network links (Lafferty, Dobson, & Kuris, 2006). Thus, the study of host–symbiont ecology is vital to understand many important processes, such as emerging infectious diseases (Hoberg & Brooks, 2015), biological invasions (Traveset & Richardson, 2014), crop pests (Hosokawa, Kikuchi, Shimada, & Fukatsu, 2007) or the effect of climate change upon biodiversity (Carlson et al., 2017). Historically, most efforts have been directed to the study of parasites with direct harmful effects on humans or livestock. Symbiont systems involving commensals and mutualists are relatively poorly studied compared to free‐living organisms and host–parasite systems (Jovani, Doña, Labrador, & Serrano, 2017).

Host–symbiont interactions rarely involve a simple one‐symbiont: one‐host interaction. Rather, even without considering the interaction of the host species with other free‐living species, any host–symbiont interaction typically involves several other species (Hopkins, Wojdak, & Belden, 2017; Poulin, 2010). In addition, whether a particular symbiont species acts as a parasite, commensal or mutualist can be highly context‐dependent (i.e., the mutualism–parasitism continuum framework; for example, Brown, Creed, Skelton, Rollins, & Farrell, 2012; Cheney & Côté 2005; Newton, Fitt, Atkins, Walters, & Daniell, 2010; Jovani et al., 2017). Thus, the study of symbionts as a whole, and not separately according to the presumed nature of their relationships with their hosts, is needed (Jovani, 2003; Jovani et al., 2017).

Defensive mutualisms (i.e., those in which symbionts protect their hosts from natural enemies, which have been often perceived as biological curiosities) have been reviewed recently following this approach and placed into this framework (Hopkins et al., 2017). Accordingly, defensive mutualisms, instead of being anecdotal host–symbiont associations, have been revealed as diverse and common associations in a wide range of plants and animal hosts from nearly all habitats on the planet. Nonetheless, with a few exceptions, most of the diversity of host–symbiont associations remains unexplored or largely unstudied.

A good example of our lack of knowledge of these interactions involves symbiotic relationships between birds and their feather mites (Acariformes: Astigmata: Analgoidea and Pterolichoidea). These mites are the most abundant and diverse ectosymbionts of birds. Almost all bird species harbour species‐ or genus‐specific feather mites (Doña, Proctor, Mironov, Serrano, & Jovani, 2016; Gaud & Atyeo, 1996; Proctor, 2003). Feather mites are highly specialized symbionts due to their (i) life cycle (i.e., they are permanent ectosymbionts, Dabert & Mironov, 1999; Proctor, 2003); (ii) high host specificity (Doña, Proctor, Mironov, Serrano, & Jovani, 2017); (iii) specific distribution on particular feathers and microsites on feathers (Fernández‐González, Pérez‐Rodríguez, de la Hera, Proctor, & Pérez‐Tris, 2015; Jovani & Serrano, 2001, 2004; Stefan et al., 2015); and (iv) mainly vertical mode of transmission (Doña, Potti, et al., 2017; Jovani, Tella, Sol, & Ventura, 2001; Mironov & Malyshev, 2002). However, as with many other symbionts, they are challenging to study, and this has strongly hampered our comprehension of this system (Doña, Diaz‐Real, et al., 2015; Proctor, 2003; Proctor & Owens, 2000).

A long‐standing question in understanding the interaction between feather mites and birds is whether these mites feed on bird tissues (e.g., feathers, skin, blood) or upon resources found on the bird's surface (e.g., algae, fungi). If they feed on bird tissues, they are more likely to be classified as parasites (Harper, 1999; Poulin, 1991; Thompson, Hillgarth, Leu, & McClure, 1997), while if they do not, feather mites would more likely be commensals or even mutualists (Blanco, Tella, & Potti, 1997; Blanco, Tella, Potti, & Baz, 2001; Galván et al., 2012). Previous evidence has suggested that feather mites could feed mainly on the uropygial gland oil of birds (Dubinin, 1951; Proctor, 2003; Walter & Proctor, 2013c). However, this oil is a nitrogen‐deficient source (Jacob & Ziswiler 1982; Proctor, 2003), and previous evidence has shown that feather mites complement their diet with fungi, pollen and algal particles (Blanco et al., 2001; Dubinin, 1951; Proctor, 2003; Walter & Proctor, 2013c). Examining thousands of slide‐mounted feather mites from 26 mite species, Dubinin (1951) found that almost all mite species had fungal spores in their guts, most from *Cladosporium*,* Alternaria* and rust fungi. Moreover, Blanco et al. (2001) found fungal mycelia and spores in the guts of 53% of *Pterodectes rutilus* (Robin) (Proctophyllodidae) and 38% of *Scutulanyssus nuntiaventris* (Berlese) (Pteronyssidae) mites from two species of swallows (Hirundinidae). Likely because of this potential mixture of feather mite diet, a recent isotopic study (Stefan et al., 2015) of the diet of two feather mite species produced inconclusive results. Interestingly, however, this study showed a strong correlation between the isotopic carbon signatures among mites inhabiting the same individual host, and between the carbon signature (but not the nitrogen signature) of feather mites and the blood of their individual bird host, thus suggesting that diet could be mainly based on shared host‐associated resources, arguably preen gland oil (Stefan et al., 2015). Thus, it remains an open question to what extent feather mites feed on uropygial oil or also upon other bird tissues, whether exogenous resources, such as fungi and bacteria, constitute an important food resource for these mites, and which specific taxa are eaten by feather mites.

In this study, we investigated the diet of feather mites using two complementary methods. First, we used light microscopy to examine feather mite gut contents under the microscope from a large sample of feather mites from ~ 200 bird species. Light microscopy allows detection of feather fragments, fungi, plant material and algae that are refractory to the clearing and mounting media (see Materials and methods). In a second approach, for a smaller number of vane‐dwelling mite species, we studied gut contents using high‐throughput sequencing (HTS) and DNA metabarcoding. This molecular approach complemented the light microscope analysis for certain potential food resources that would not be easily recognized in the slide‐mounted specimens (e.g., bacteria, soft bird tissues) and also allowed for a detailed analysis of fungi, bacteria and plant taxa in the mites’ diet.

## 2 MATERIALS AND METHODS

### 2.1 Gut content assessment via light microscopy

For the microscopy analysis, we used previously slide‐mounted mites from the Proctor Lab collection of feather mites from around the world. Mites had been cleared in lactic acid and mounted in polyvinyl alcohol medium (#6371A; BioQuip, Rancho Dominguez, CA, USA). This process clears soft tissues but retains refractory material (e.g., chitin, cellulose). Selection of mites to examine was based on taxonomic diversity of mites and host birds, and ecological breadth of hosts (e.g., birds from terrestrial, marine and freshwater habitats, including predators, granivores, nectarivores, etc.). We initially examined several thousand mites using a Leica DMLB compound microscope with DIC lighting. Mites with visible gut contents were photographed at various magnifications (200, 400 and 800×) depending on size of material in the gut. For each host bird species included in the study, our goal was to photograph a minimum of five individual mites from each mite genus present on the bird species. In some cases, if there were fewer than five mites with gut contents available for a mite genus and/or bird species, then all the available mites that contained gut contents were photographed. Under ideal circumstances, we would have focused on mite species rather than genera, but particularly for tropical areas, feather mite alpha‐taxonomy is in an early state and many species have yet to be described. Also, for many taxa, only adult males can be readily ascribed to species, and we wished to include nymphal and female mites in our assessment. Mites were identified to genus using Gaud and Atyeo (1996) with additional literature for more recently described genera (e.g., Valim & Hernandes, 2010). In total, 1,300 individual mites representing 100 genera (18 families) from 190 host bird species (72 families; 19 orders) were photographed.

Each morphologically unique type of gut content was given a code, and for every individual mite, all the types of gut content present were recorded, as well as the approximate amount of each type of gut content. Aided by illustrations in Lacey and West (2006) and consultation with a mycologist (T. Spribille, University of Alberta), we then classified all unique types of gut contents as fungi, diatoms, plant spores, “unidentifiable” and oily globules (possibly uropygial gland oil or digestive by‐products in peritrophic membranes). Unidentifiable objects were mainly extremely small fragments or flecks of material <5 μm long (some of which could have potentially been tiny remnants of feather barbules) (e.g., Figure S10). Oil globules were not included in the analyses, as we consider that our ability to consistently identify this material was much lower than for other types of gut content (see an example of potential oil globs in Figure S11).

### 2.2 Sample collection and sterilization for DNA metabarcoding

For the DNA metabarcoding study, 1,833 individual mites of 18 mite species from 18 passerine bird host species (34 individual birds or infrapopulations) were sampled from birds captured with mist nets in Andalusia (Spain) during the spring of 2015 (see Table S1, for sampling details). An effort was made to collect all mites found on the wing flight feathers from each sampled bird, using a sterile swab impregnated with ethanol. Mites were preserved at −20*°*C in tubes with 96% ethanol. In those cases in which more than one mite species was found on an individual bird, one different sterile swab was used for collecting each tentative mite species (according to Doña et al., 2016 based on genus‐specific location on bird feathers) into different tubes.

Mites were sterilized in AllGenetics & Biology, SL (A Coruña, Spain) with three ethanol washes following Andrews (2013). Each time, tubes containing mites were agitated manually. Then, all ethanol was collected with the pipette using a thin pipette tip, with careful visual checks to avoid removing any mites. Tubes were then refilled with ethanol. Washed mites were then used for further analyses (hereafter mite samples) and the ethanol extracted from the first wash was used as the environmental control sample (hereafter, external sample).

### 2.3 DNA extraction, amplification, library construction and sequencing

DNA isolation, amplification and library preparation were carried out at AllGenetics & Biology, SL (A Coruña, Spain). Genomic DNA was extracted from each mite sample using the HotSHOT method (Truett et al., 2000). Briefly, the ethanol from the last mite wash was evaporated and a 1‐M NaOH solution was added to the dried wells, incubated at 95*°*C and neutralized with equivalent amounts of Tris–Cl. The final extraction volume was 30 μl. A negative control that contained no sample was included in every extraction round to check for contamination during the experiments. This procedure preserves exoskeletons for morphological identifications (see Doña, Diaz‐Real, et al., 2015). However, in contrast to more aggressive isolation methods, DNA from Gram‐positive bacteria, undigested diatoms and intact fungal spores may not have been amplified. After DNA extraction, the remaining exoskeletons were separated from the buffer and stored in 80% ethanol. External samples were extracted as follows. The ethanol phase from the first mite wash was pipetted onto a nitrocellulose filter (ca. 9 cm² with a pore size of 22 μm), and then, DNA was isolated using the PowerSoil DNA isolation kit (Mobio) following manufacturer's instructions. The final elution volume was 50 μl.

From each sample, a total of seven libraries were built: five from DNA extracted from mite samples and two from the DNA extracted from the external samples (i.e., see above for sample name definitions). HTS libraries were prepared by amplifying a different molecular marker and by adding the Illumina‐specific sequencing primers, indices and adaptors. The regions amplified from mite samples were as follows: the bacterial/archaeal 16S rRNA gene variable region 4 (515F/806R, Caporaso et al., 2012), the ITS 2 region of the fungal rRNA operon (ITS86F/ITS4, De Beeck et al., 2014), the ITS 2 region of plants and algae (S2F/S3R, Chen et al. 2010) and the region of the mitochondrial COI gene of birds. To maximize the potential for retrieving bird DNA, we used internal primers of the mitochondrial COI gene suitable for amplifying degraded DNA (BirdF1/AvMiR1, Kerr, Lijtmaer, Barreira, Hebert, & Tubaro, 2009). In addition, we amplified the COI gene of feather mites (bcdF05/bcdR04, Dabert, Ehrnsberger, & Dabert, 2008) to molecularly confirm the mite species identity (Doña, Diaz‐Real, et al., 2015). Only bacterial and fungal regions were amplified from the external samples.

Libraries were built following the recommended protocol by Illumina for bacterial 16S metabarcoding, with some modifications. Similar protocols have been used by other authors (e.g., Lange et al., 2014; Vierna, Doña, Vizcaíno, Serrano, & Jovani, 2017). Briefly, the libraries were constructed in a two‐step PCR (hereafter, PCR1 and PCR2): PCR1s were carried out in a final volume of 25 μl, containing 6.50 μl of Supreme NZYTaq Green PCR Master Mix (NZYTech), 0.5 μM of each primer and PCR‐grade water up to 25 μl. Thermal cycling conditions included an initial denaturation step at 95*°*C for 5 min, followed by 35 cycles of denaturation at 95*°*C for 30 s, annealing at various temperatures (bacteria: 50*°*C; fungi: 52*°*C; plant: 51*°*C; bird: 59*°*C; mite: 55*°*C), extension at 72*°*C for 45 s and a final extension step at 72*°*C for 10 min. PCR1 products were purified by solid‐phase reversible immobilization (SPRI) (Hawkins, O'Connor‐Morin, Roy, & Santillan, 1994), using Mag‐Bind RXNPure Plus magnetic beads (Omega Biotek). To eliminate the primer dimers generated during PCR, we used a final bead concentration of 0.5X, thus size selecting the high molecular weight amplicons over primer dimers. The purified products were loaded in a 1% agarose gel stained with GreenSafe (NZYTech) and visualized under UV light.

PCR2 was carried out using 2.5 μl of the amplified DNA from PCR1 as a template and was performed under the same conditions as PCR1, but only running five cycles at 60*°*C as the optimal annealing temperature.

A total of 31 different index combinations were used, and 40 PCR cycles were performed (Vierna et al., 2017). The resulting products were purified following the SPRI method as indicated above. Likewise, the purified products were loaded in a 1% agarose gel stained with GreenSafe (NZYTech) and visualized under UV light.

All products (a total of 238 libraries) were pooled together in 21 sets of differentially indexed samples. All pools were quantified with Qubit^™^ fluorometer (Invitrogen). We did not obtain bird DNA in any sample and plant DNA only from two samples (see Results below). Accordingly, all except one plant pool (i.e., the one containing the only two samples successfully amplified, see Results below) were not sequenced as they did not reach the minimum amount of DNA for HTS.

All pools were sequenced by Novogene (Beijing, China) on Illumina HiSeq 4000 using the PE 250 strategy (see Supporting Information for coverage information; Table S2). Quality controls were carried out using company in‐house Perl scripts to remove contaminated adaptors and low‐quality sequences.

### 2.4 Bioinformatic analysis

Bacterial sequences were postprocessed and classified with mothur v1.38.1 (Schloss et al., 2009) according to the MiSeq SOP (accession date: 30 August 2016, Kozich, Westcott, Baxter, Highlander, & Schloss, 2013). In brief, sequences were aligned and classified against the silva (v123) database (Pruesse et al., 2007). Potential mitochondrial, chloroplastidial and other nontarget sequences were removed, and the UCHIME algorithm was used to identify and remove chimeras (Edgar, Haas, Clemente, Quince, & Knight, 2011). Lastly, sequences were clustered into OTUs using the *cluster.split* command. Fungal sequences were processed using the PIPITS pipeline (Gweon et al., 2015). Briefly, this procedure extracts the ITS subregion from reads and then assigns them taxonomically with a trained RDP Classifier (Bengtsson‐Palme et al., 2013). One mite sample containing <100 reads after preprocessing was not used for further analyses on fungal sequences (see Table S2). Plant raw reads were quality trimmed (sliding window of 30 bp with a minimal average Phred score of 33) using trimmomatic 0.36 (Bolger, Lohse, & Usadel, 2014) and then clustered to OTUs at 97% using cd‐hit version 4.5 (Fu, Niu, Zhu, Wu, & Li, 2012). Representative (centroid) sequences were blasted using megablast against the ncbi “nr” nonredundant nucleotide sequence collection (National Center for Biotechnology Information: http://www.ncbi.nlm.nih.gov/).

Mite identity was molecularly confirmed in all cases using a similar pipeline to that used in Doña, Moreno‐García, Criscione, Serrano, and Jovani (2015). In brief, we used Geneious R10 (http://www.geneious.com, Kearse et al., 2012) plugin *Sequence classifier*, over a concatenated file containing the forward and reverse reads (quality trimmed as described above for plant libraries and with a minimum length of 200 bp). Then, we used the recommended threshold and a reference DNA barcode library (Doña, Diaz‐Real, et al., 2015).

### 2.5 Statistical analysis

Differences in prevalence and morphological diversity of diet resources (the maximum diversity retrieved for each mite sample, that is, each mite infrapopulation; see above) from microscopy assessments were analysed using generalized linear mixed models (GLMM) (glmer function from package lme4 1.1‐12, Jovani & Tella, 2006; Bates, Mächler, Bolker, & Walker, 2015). For assessing differences in prevalence, we ran a binomial GLMM considering prevalence (1: presence, 0: absence) as the response variable, the type of food resource as the predictor variable and the bird infrapopulation nested into bird species plus mite genera as random factors. For assessing differences in morphotype diversity of fungi and diatoms, we ran a Poisson GLMM considering morphotype diversity as the response variable, and the same structure of predictor and random factors. We confirmed assumptions underlying GLMMs by exploring regression residuals for normality against Q‐Q plots.

Fungal and bacterial OTUs were imported to r and manipulated using phyloseq r package (McMurdie & Holmes, 2013). In particular, we studied the variance in bacterial and fungal assemblage composition among infrapopulations using a permutational multivariate analysis of variance on Bray–Curtis and Jaccard distance matrices (PERMANOVA; *adonis* function from the vegan v2.4.1 r package, Oksanen et al., 2017). The null hypothesis was that the centroid does not differ between host species and/or mite species (Anderson & Walsh, 2013). This test is highly sensitive to data dispersion (Anderson, 2001), and thus, we tested it with the multivariate homogeneity PERMDISP2 procedure (Anderson, 2006; *betadisper* function from vegan, Anderson & Walsh, 2013) with 999 permutations. Additionally, following previous approaches to overcome this statistical issue (e.g., Brice, Pellerin, & Poulin, 2017), we explored the community clustering with ordination analyses (principal coordinates analyses, PCoA) and stacked bar plots at the infrapopulation level.

## 3 RESULTS

### 3.1 Composition and morphological diversity of feather mites’ diets assessed by microscopy

**Figure 1:**
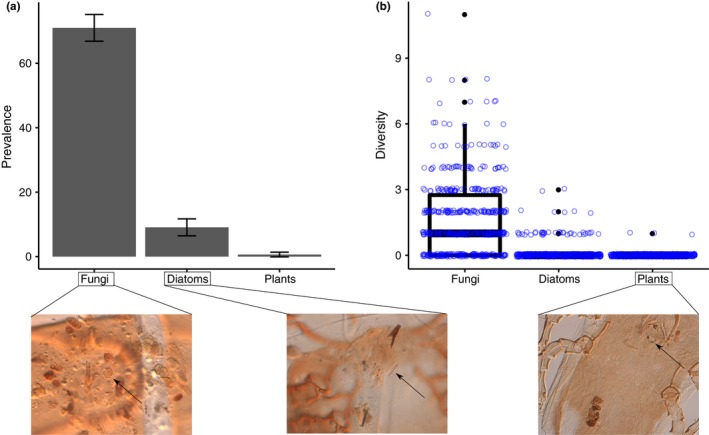
Barplot and boxplot depicting the (a) prevalence (*N* = 481) and (b) morphological diversity (using the maximum diversity retrieved per infrapopulation) of diet items found in the microscopy assessment of feather mite gut contents. Error lines in (a) represent confidence intervals (95%). Blue dots in (b) represent real data points (jittered). Representative pictures of each food resource are placed beneath the plots [Colour figure can be viewed at http://wileyonlinelibrary.com]

**Figure 2:**
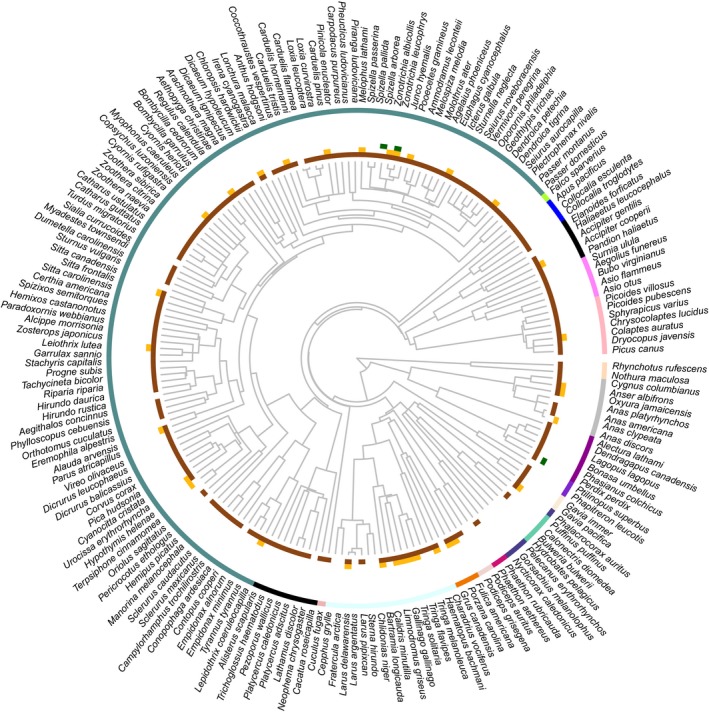
50% majority‐rule consensus phylogenetic tree depicting the distribution of food resources retrieved by microscopic analysis of feather mite gut contents across the phylogeny of birds. In brief, 1,000 trees were obtained from BirdTree (Jetz, Thomas, Joy, Hartmann, & Mooers, 2012, http://birdtree.org) and summarized using sumtree v 4.1.0 in dendropy v4.1.0 (Sukumaran & Holder, 2010, 2015), following Rubolini, Liker, Garamszegi, Møller, and Saino (2015). Rings from the centre out, brown: fungi. Mustard: diatoms. Green: plants. Most external ring colours depict bird orders [Colour figure can be viewed at http://wileyonlinelibrary.com]

From a total of 481 infrapopulations (1,300 individual mites) belonging to 190 bird species and 100 mite genera, fungal material (spores and hyphae) was the most prevalent type of gut content (GLMM: χ*²* = 168.73, *df* = 2, *p* < .001; Figure 1) and the most morphologically diverse (GLMM: χ*²* = 442.5, *df* = 2, *p* < .001; Figure 1). In addition, diatoms and plant material were also found, but in a much lower frequency and morphotype diversity than fungi (Figure 1). Highly similar results were found when only analysing passerines (Figure S1 and S2), the avian order in which bird species were also studied using DNA metabarcoding (see below). The overall predominance of fungi was widespread across the avian phylogeny (Figure 2) and feather mite taxonomy (Table 1).

**Table 1:**
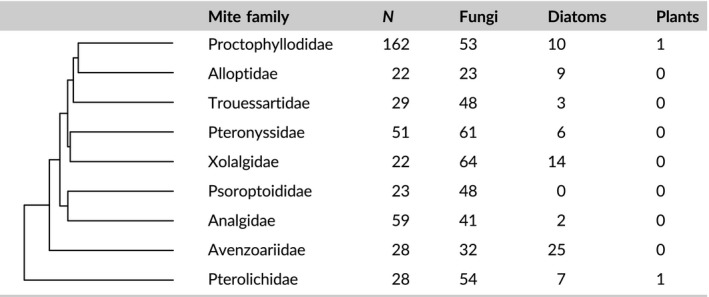
Prevalence (% of feather mite infrapopulations) of identified food items found in the best‐sampled mite families. Phylogenetic information was retrieved from Klimov and O'Connor (2013)

### 3.2 DNA metabarcoding of feather mites’ diets

**Figure 3:**
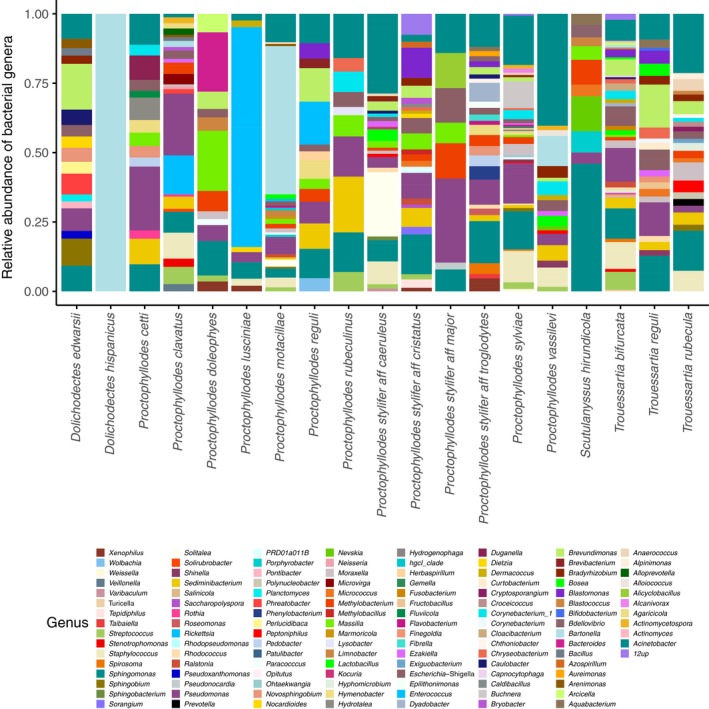
Stacked bar plots of the bacterial genera retrieved in the molecular analyses of mite species. Low abundance taxa (<2%) were not shown for illustrative purposes [Colour figure can be viewed at http://wileyonlinelibrary.com]

**Figure 4:**
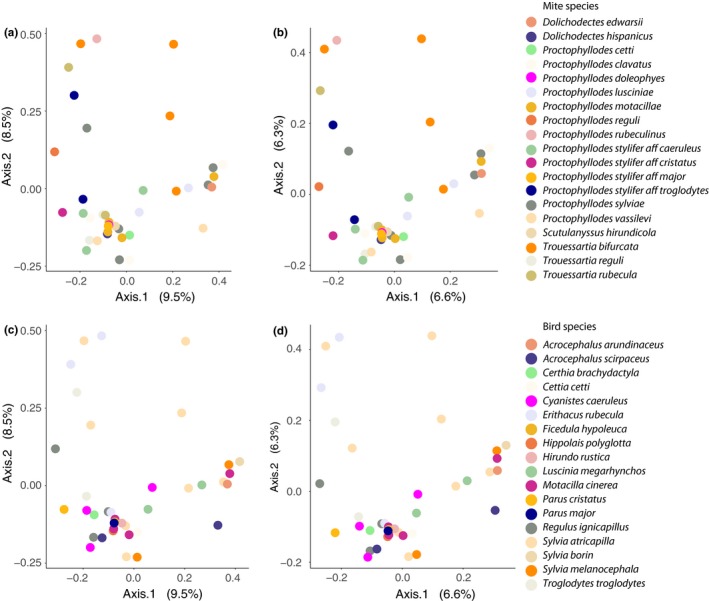
Principal coordinates analysis (PCoA) of bacterial communities of feather mite infrapopulations: First row, samples coloured by mite species and (a) based on Bray–Curtis and (b) Jaccard distances, respectively; Second row, samples coloured by bird species and c) based on Bray–Curtis and (d) Jaccard distances, respectively. OTUs counts were scaled to the smallest library following McMurdie and Holmes (2014) and Denef, Fujimoto, Berry, and Schmidt (2016) [Colour figure can be viewed at http://wileyonlinelibrary.com]

Metabarcoding results of the mite species from the genera Proctophyllodes Robin, 1877, Trouessartia Canestrini, 1899, Dolichodectes Park & Atyeo, 1971, and Scutulanysuss Mironov, 1985 showed highly congruent results with the microscopic analyses in terms of the prevalence and diversity of food resources, while complementing them with bacterial detection and providing taxonomic detail of the organisms involved. We found bacterial DNA in all samples (Table S2). The bacterial genera identified primarily belonged to the phyla Proteobacteria, Actinobacteria and Bacteroidetes, with Proteobacteria being the most frequently represented (Figure S5). Within these phyla, we retrieved a high diversity of bacterial genera (Figures 3, S7 and S8). Genera commonly found in soil and as environmental “background noise” such as Sphingomonas, Acinetobacter and Pseudomonas were the most prevalent genera (Table 2, Figures 3, S7 and S8) while typically endosymbiotic genera such as Bartonella, Enterococcus and Buchnera were the most abundant when they were present (Table 2, Figures 3, S7 and S8). PERMANOVAs showed statistically significant differences in bacterial composition between mite (53% variance, *F* = 1.25, *p* = .006) and bird species (52% variance, *F* = 1.31, *p* = .001). Nonetheless, we found different levels of dispersion between mite (*F* = 7.19, *p* = .001) and bird species (*F* = 9.95, *p* = .001). In addition, ordinations as well as individual stacked bar plots of bacterial profiles did not show clustering by mite or by bird species in bacterial OTUs or genera (Figures 4 and S7). Additionally, a re‐analysis excluding all bacterial OTUs found in the external samples, that is, to exclude potential environmental contamination coming from bacterial OTUs still remaining after mite washes, showed almost identical results: significant differences in bacterial composition between mite species (PERMANOVA, 51% variance, *F* = 1.15, *p* = .023) and bird species (PERMANOVA, 49% variance, *F* = 1.20, *p* = .01). Nonetheless, again, we found different levels of dispersion between mite species (*F* = 8.46, *p* = .002) and between bird species (*F* = 11.84, *p* = .001). In addition, ordination and profile plots did not show clustering by either mite or bird species in bacterial OTUs and genera (Figures S8 and S9).

**Table 2:** Prevalence and abundance (mean; minimum–maximum) statistics from the 30 most prevalent fungal and bacterial genera retrieved by DNA metabarcording. The three genera which were, on average, most abundant for each taxon, are asterisked and highlighted in bold. Relative abundance was calculated as the % of sequences of the given genus in those samples where the genus was found

Fungi	Prevalence (% of samples)	Relative abundance (% sequences within samples)	Bacteria	Prevalence (% of samples)	Relative abundance (% sequences within samples)
*Cladosporium*	63	17; 2–62	*Sphingomonas*	88	12; 5–33
*Toxicocladosporium*	63	26; 2–89	*Acinetobacter*	71	18; 5–66
*Aureobasidium*	53	26; 2–70	*Pseudomonas*	71	14; 5–50
*Cryptococcus*	42	6; 2–11	*Sediminibacterium*	53	10; 6–19
*Malassezia	**42**	**31; 3**–**94**	*Brevundimonas*	47	11; 6–18
*Penicillium*	42	11; 2–43	*Escherichia*–*Shigella*	41	7; 5–12
*Rhodotorula*	32	7; 2–21	*Staphylococcus*	41	14; 5–35
*Acremonium*	26	9; 2–18	*Methylobacterium*	35	8; 6–12
*Catenulostroma*	26	13; 3–37	*Massilia*	29	10; 6–21
*Devriesia*	26	7; 2–14	*Bartonella	**24**	**42; 6**–**90**
*Erysiphe*	26	23; 7–76	*Blastomonas*	24	9; 5–14
*Pleurotus*	26	8; 2–13	*Streptococcus*	24	10; 6–13
*Alternaria*	21	13; 6–18	*Bradyrhizobium*	18	6; 5–7
*Aspergillus*	21	10; 2–29	*Corynebacterium_1*	18	7; 7–7
*Beauveria*	21	8; 4–11	*Lactobacillus*	18	8; 6–10
*Erythrobasidium*	21	10; 2–22	*Moraxella*	18	8; 5–11
*Sporobolomyces*	21	5; 2–7	*12up*	12	11; 8–13
*Talaromyces	**21**	**30; 3**–**98**	*Actinomycetospora*	12	5; 5–6
*Dioszegia*	16	3; 3–4	*Bosea*	12	7; 6–9
*Golovinomyces*	16	13; 2–26	*Chryseobacterium*	12	6; 6–6
*Meira	**16**	**47; 5**–**73**	*Enterococcus	**12**	**40; 14**–**57**
*Phaeotheca*	16	21; 18–27	*Alicyclobacillus*	6	12; 12–12
*Pseudocercospora*	16	15; 12–21	*Anaerococcus*	6	7; 7–7
*Stagonospora*	16	11; 2–27	*Aquabacterium*	6	6; 6–6
*Tilletiopsis*	16	6; 3–8	*Arcicella*	6	6; 6–6
*Arthrocatena*	11	3; 3–3	*Bacteroides*	6	20; 20–20
*Claviceps*	11	4; 3–6	*Buchnera	**6**	**41; 41**–**41**
*Debaryomyces*	11	17; 4–29	*Cloacibacterium*	6	9; 9–9
*Exobasidium*	11	11; 9–14	*Duganella*	6	8; 8–8
*Farysizyma*	11	8; 5–12	*Dyadobacter*	6	10; 10–10

**Figure 5:**
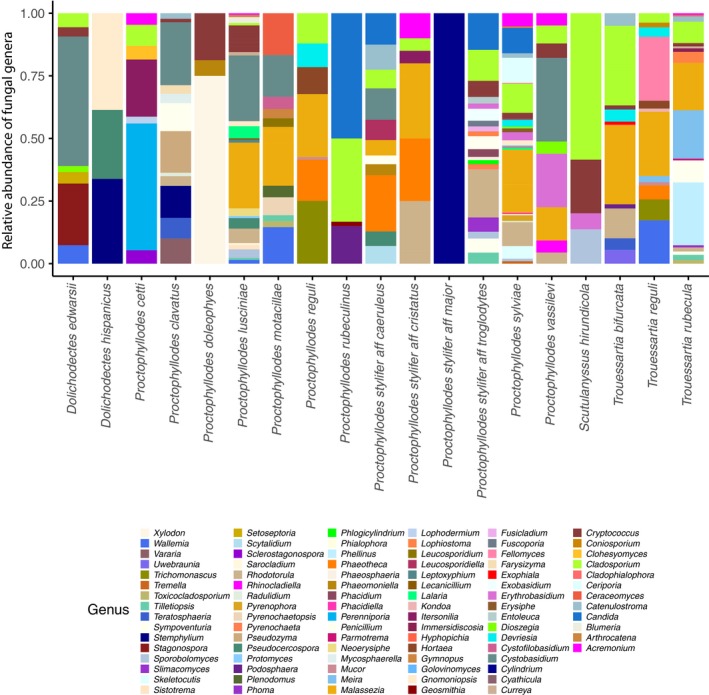
Stacked bar plots of the fungal genera retrieved in the molecular analyses of mite species. Low abundance taxa (<2%) were not shown for illustrative purposes [Colour figure can be viewed at http://wileyonlinelibrary.com]

**Figure 6:**
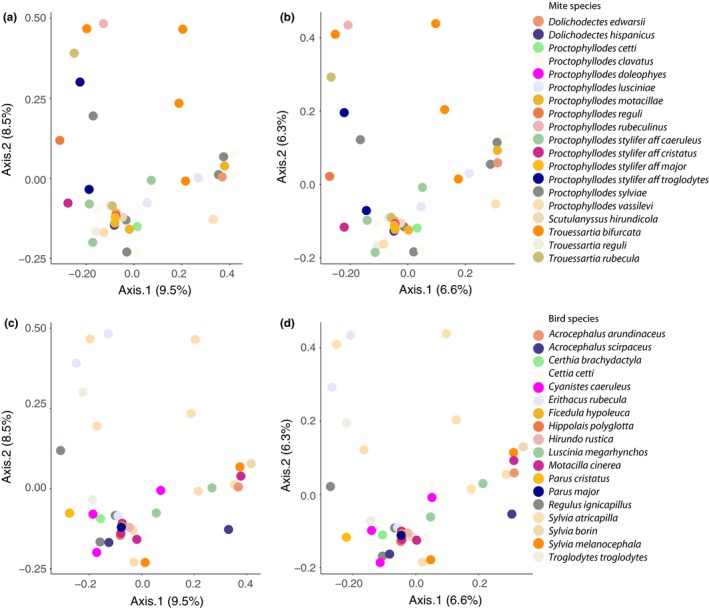
Principal coordinates analysis (PCoA) of fungal communities of feather mite infrapopulations: First row, samples coloured by mite species and (a) based on Bray–Curtis and (b) Jaccard distances, respectively; second row, samples coloured by bird species and (c) based on Bray–Curtis and (d) Jaccard distances, respectively. OTUs counts were scaled to that of the smallest library following McMurdie and Holmes (2014) and Denef et al. (2016) [Colour figure can be viewed at http://wileyonlinelibrary.com]

We found fungal DNA in all infrapopulations except one (Table S2). Overall, we retrieved a high diversity of fungal species, which was much higher in the mite samples compared to the external samples (See Material and Methods above, Figure S5). Fungal species retrieved from mite samples mostly belonged to the phyla Ascomycota and Basidiomycota, with Ascomycota being the most represented (Figure S4). At the genus level, the most prevalent were Cladosporium, Toxicocladosporium and Aureobasidium (Table 2, Figures 5 and S6). On the other hand, Meira, Malassezia and Talaromyces were the most abundant fungal genera when present (Table 2, Figures 5 and S6). Interestingly, we retrieved genera for which keratinolytic activity is known, such as Cladosporium, Acremonium, Malassezia, Penicillium and Phoma. PERMANOVAs showed significant differences in fungal composition between mite species (51% variance, *F* = 1.18, *p* = .027) and bird species (49% variance, *F* = 1.21, *p* = .016). Nonetheless, dispersion analyses (see Methods) revealed different levels of dispersion between mite species (*F* = 9.22, *p* = .004) and between bird species (*F* = 9.36, *p* = .002), suggesting the need for a detailed inspection of the within‐species variance. By doing so, principal coordinates analyses as well as stacked bar plots at the individual level within species showed no apparent consistency of fungal profiles either within mite or bird species (Figures 6 and S6).

Plant DNA was only sequenced from two infrapopulations (of 34) from two mite species inhabiting two different bird individuals. The first infrapopulation from which plant DNA was recovered belonged to *Proctophyllodes sylviae* Gaud, 1957 from the Blackcap *Sylvia atricapilla* (Linnaeus, 1758). Plant OTUs retrieved matched to *Polygala teretifolia* Thunb. (99.7% pairwise similarity; grade 88.6%), *Citrus clementine* hort. (two OTUs: 92.8, 98.4% pairwise similarity; grade 96.4, 99.2%), *Daphne laureola* L. (94.9% pairwise similarity; grade 93.2%) and *Digitalia ciliaris* (Retz.) Koeler. (96.5% pairwise similarity; grade 96%). The second infrapopulation belonged to *Trouessartia bifurcata* (Trouessart, 1885) also from a *Sylvia atricapilla* host, in which the single OTU retrieved matched to *Quercus* sp. (95.5% pairwise similarity; grade 97.7%).

## 4 DISCUSSION

In this study, by analysing the diet of feather mites using both DNA metabarcoding and microscopy‐based methods, we investigated the long‐standing question of the nature of the interaction between birds and feather mites. Fungi and potentially bacteria (see below) were revealed as the main recognizable food resources for feather mites, while diatoms and plant matter appeared as rare food resources. Similarly, Dubinin (1951) examined the guts of 18,735 specimens of *Freyana* spp. (Freyanidae) from waterfowl and found diatoms in only 135 of them (0.72%). Importantly, we did not find visual or DNA evidence of feather mites feeding upon bird resources (e.g., blood, skin) other than likely uropygial gland oil (see Materials and Methods), in spite of using primers suitable for amplifying degraded bird DNA. We observed no obvious feather filaments in our microscopy analysis, but this and our molecular study would not have been able to identify tiny (non‐DNA‐bearing) fragments of feathers, which have been occasionally reported in microscopy studies. The chelicerae of vane‐dwelling feather mites do not seem capable of cutting or tearing intact feathers, so if the tiny fragments we observed in the guts are indeed feather fragments, they would likely be ingested along with other loose material. In addition, we found a high prevalence of both keratinophilic and pathogenic fungal taxa (e.g., *Cladosporium*,* Penicillium,* Al Rubaiee, Al Murayati, Nielsen, & Møller, 2017; Friedrich, Gradišar, Mandin, & Chaumont, 1999; Gunderson, 2008; Marchisio, Curetti, Cassinelli, & Bordese, 1991; Nwadiaro, Ogbonna, Wuyep, & Adekojo, 2015) in feather mite guts. Whether the quantities of bacteria and fungi eaten by feather mites are enough to increase host fitness requires further study. Altogether, our results support previous evidence on the commensalistic–mutualistic role of vane‐dwelling feather mites (Blanco et al., 1997, 2001; Galván et al., 2012; Proctor, 2003; Walter & Proctor, 2013a,b,c). Thus, vane‐dwelling feather mites probably should no longer be considered to be parasites of birds (e.g., Harper, 1999) but rather commensalists–mutualists. This does not apply to the few taxa of quill‐dwelling feather mites that clearly feed on feather pith (e.g., Ascouracaridae) or those that live on or in the epidermis of the host (e.g., Dermationidae, Epidermoptidae) (Gaud & Atyeo, 1996; Proctor, 1999). Additionally, whether uropygial gland oil constitutes an important food resource for feather mites remains unanswered from our data (Pap, Vágási, Osváth, Mureşan, & Barta, 2010) and should be studied using more sensitive methods (e.g., HPLC, histological staining analysis). Indeed, should uropygial gland oil be beneficial for birds, a large number of mites feeding upon this resource might have a detrimental effect on host fitness (Blanco et al., 2001). However, a recent review concluded that is not even clear how or if uropygial gland oil affects bird fitness (Moreno‐Rueda, 2017). In the light of our findings, previous occasional documentation of unhealthy birds with high numbers of vane‐dwelling feather mites (e.g., Atyeo & Gaud, 1979) could be reinterpreted as birds in poor condition providing more food resources to feather mites (e.g., fungi and bacteria, which may be directly or indirectly related with host’ health status, Blanco et al., 2001; Soler et al., 2012). It may also be that birds in poor condition preen less, which could in turn impact the abundance of feather mites if they are susceptible to removal by preening activities. However, it remains the possibility that feather mites have an effect on host fitness by removing preen gland oil, by potential aerodynamic costs of harbouring large amounts of mites and by indirect effects on host fitness mediated by other ectoparasites (e.g., the occasional ingestion of feather mites by feather lice which may indirectly increase the cost of parasitism of feather lice).

The possibility that symbiont species might be at risk of extinction (e.g., Carlson et al., 2017; Rózsa & Vas, 2014) suggests the need for a rapid integration of this knowledge into bird‐related practices, such as those in wild bird conservation programmes. Also, our results suggest that further studies of birds in farms, zoos and the pet trade are needed, where traditionally feather mites were viewed as parasites, with birds provided with treatment using acaricides (e.g., Alekseev, 1998; Salisch, 1989). This practice not only has the downside of monetary expense because of the use of acaricides, but could also result in the loss of the potential services provided by feather‐cleaning mites, as our results suggest.

Analyses of the bacterial and fungal DNA found in the guts of feather mites revealed a high diversity of taxa that were not structured by host or by mite species (Figures 4, 6 and S6‐S9). This suggests trophic opportunism of mites (da Silva, Dorrestein, & Quinn, 2015; Kent & Burtt, 2016), which may graze upon whatever food resources might be available at the time. This opportunistic “feather‐cleaning” feeding behaviour is also supported by the large amount of unidentifiable items we found in the guts and by the higher abundance and diversity of fungi found in the mite samples in comparison with the external samples (e.g., Figures S3 and S10). Overall, many other species of sarcoptiform mites, including many free‐living Astigmata, are functionally defined as fungivore–microbivore–detritivores (e.g., Pyroglyphidae and most oribatid mites, Walter & Proctor 2013a,b), and our results also support this classification for feather mites. In fact, our results are in large agreement with previous studies on microbes found in other mite species (Chaisiri, McGarry, Morand, & Makepeace, 2015; Hubert et al., 2012), where strong evidence has been found for the utilization of bacteria as a food source in free‐living astigmatan species (Erban & Hubert, 2008, 2010; Hubert, Nesvorna, Kopecký, Ságová‐Marečková, & Poltronieri, 2014; Hubert et al., 2016). In these studies, microbiomes composed of highly diverse taxa in low abundance have been interpreted as evidence for microbivory. In contrast, microbiome profiles showing a low diversity of highly abundant taxa are interpreted as evidence of symbiotic or pathogenic bacterial species (Hammer, Janzen, Hallwachs, Jaffe, & Fierer, 2017; Hubert et al., 2016). In this way, the prevalence–abundance patterns of the bacteria found here (Table 2) suggest a combination of bacteria used as food resource (mostly environmental‐associated genera, which were more prevalent but less abundant, *for example*,* Sphingomonas* and *Acinetobacter*; Table 2) and of potentially symbiotic, commensalistic or pathogenic bacteria (less prevalent but much more abundant when present, *for example*,* Bartonella*,* Enteroccocus;* and the primary endosymbiont, *Buchnera*; Table 2).

Lack of a stable “microbiome” across different individuals of a given species has been found in other organisms with a nutritionally broad diet (Shapira, 2016). In contrast, species with highly biased diets, such as lice feeding on bird feathers (mainly keratin) or termites feeding on dead wood (mainly cellulose), typically have permanent and relatively stable endosymbiotic bacteria which provide them essential vitamins or other nutritional supplements (Puchta, 1955; Ohkuma, 2008; Perotti, Kirkness, Reed, & Braig, 2009; Boyd et al., 2016; but see Hammer et al., 2017). Thus, our results suggesting the lack of a stable microbiome at the mite species level add support to the hypothesis of a generalist fungivore–microbivore–detritivore diet for the feather mites reported here, instead of these resources being taken as a by‐product of a diet based mostly on uropygial oil (Engel & Moran, 2013; Sanders et al., 2017; Shapira, 2016). In fact, in 42% of the mites in which we detected any food resource, we did not see any oil globules (but see Materials and Methods) also suggesting that resource intake does not depend on oil ingestion.

A further understanding of the multilayered hologenome (i.e., to distinguish between stable–unstable, adapted–unadapted bacterial taxa, Shapira, 2016) through large‐scale microbiome‐oriented studies will help in disentangling the role of these potentially symbiotic or pathogenic bacteria of feather mites. Furthermore, whether feather mites select among available food resources (fungal preferences have been found in free‐living fungivorous Astigmata, Hubert et al., 2003; Hubert, Jarosık, Mourek, Kubatova, & Zdarkova, 2004) or do not need to rely on bacterial symbionts requires further experimental study. Lastly, a hypothesis of an “external‐rumen” mode of feeding, in which mites ingest predigested food (by bacteria), has been also supported in free‐living astigmatan mites (Hubert et al., 2014, 2016) and would be also compatible with our results.

Feather mite species are relatively host‐specific and (presumably) host‐specialized symbionts that appear to have relatively low levels of switching to new host species (Doña, Proctor, et al., 2017; Doña, Sweet, et al., 2017; Gaud 1992; Klimov, Mironov, & O'Connor, 2017; Matthews et al., 2018). These switches mostly involve closely related hosts, but major‐host switches (e.g., between bird orders) have been revealed as a major driver of their diversification (Doña, Proctor, et al., 2017). As for many other host–symbiont systems (Clayton, Bush, & Johnson, 2016; Nylin et al., 2017), understanding the (co)eco‐evolutionary scenario of host‐switching in this host–symbiont system is still in its infancy. However, the likely opportunistic diet of feather mites reported here suggests that host‐switching of feather mites would not be constrained by the extrinsic nutritional resources available on the new host (but it may be, *for example*, by feather morphology or by the bird preening efficiency; Clayton et al., 2005). Uropygial gland oil composition, however, differs between birds (Soini, Whittaker, Wiesler, Ketterson, & Novotny, 2013); and whether mites are specialized to host oil is unknown, and requires further study. Nevertheless, the fact that different bird species can harbour contrasting (and consistent) abundances of feather mites (Diaz‐Real et al., 2014; Doña, Moreno‐García, et al., 2015) suggests that, among others factors, the abundance of food resources for feather mites could strongly differ between bird species, but this also needs additional research.

Overall, this study supports the hypothesis that the interaction between birds and vane‐dwelling feather mites involves commensalism or mutualism, with feather mites acting as feather‐cleaners of birds. This opens the possibility of studying bird‐feather mites as an interesting case study of defensive symbiosis (Hopkins et al., 2017). Further experimental research is needed to unravel the likely context‐dependent (possibly even occasionally parasitic) relationship between vane‐dwelling feather mites and birds (Blanco et al., 2001). In particular, future studies should investigate the following. (i) Using appropriate and sensitive methods such as HPLC, test whether uropygial gland oil is part of the diet of feather mites. A comparative exploration of the diet of feather mites inhabiting birds with vestigial uropygial gland that produce powder down would be also useful. If uropygial oil is a large component of vane‐dwelling feather mites, it would be then important to test whether removal of the oil affects bird fitness. (ii) Investigate whether the diet of feather mites differs along the annual cycle of birds (e.g., migration, moult). (iii) Examine the potential aerodynamic costs of harbouring different quantities of feather mites. (iv) Determine effects of feather mites on host fitness as mediated by other ectosymbionts (e.g., feather lice). (v) Test whether an experimental increase in feather mites’ abundance increases, decreases or has no overall effect on host fitness. Lastly, (vi) examine whether experimental variation in feather mites abundance has a context‐dependent (e.g., under different environmental conditions) effect on host fitness over time.

## AUTHOR CONTRIBUTIONS

J.D., H.P., D.S., K.P., A.O., J.H., M.A. and R.J. conceived the study. J.D., R.J., H.P. and D.S. designed the study. J.D. analysed the data with the support of J.H., M.A. and R.J. J.D. wrote the manuscript with the help of all authors.

## Data Availability

The HiSeq raw data and the processed representative sequence files have been deposited in Figshare (https://doi.org/10.6084/m9.figshare.5729277).
