# Supplementary material for: Feather mites play a role in cleaning host feathers: New insights from DNA metabarcoding and microscopy
Source: Mol Ecol. 2018 May 3;28(2):203–18. doi: 10.1111/mec.14581 (PMC6905397; doi:10.1111/mec.14581)

Supplementary material

**Figure S1.** Boxplot depicting the morphological diversity of food resources (using the maximum diversity retrieved per infrapopulation) found in the microscopic assessment of gut contents of feather mite species inhabiting species of Passeriformes. Blue dots represent real data points (jittered).

**
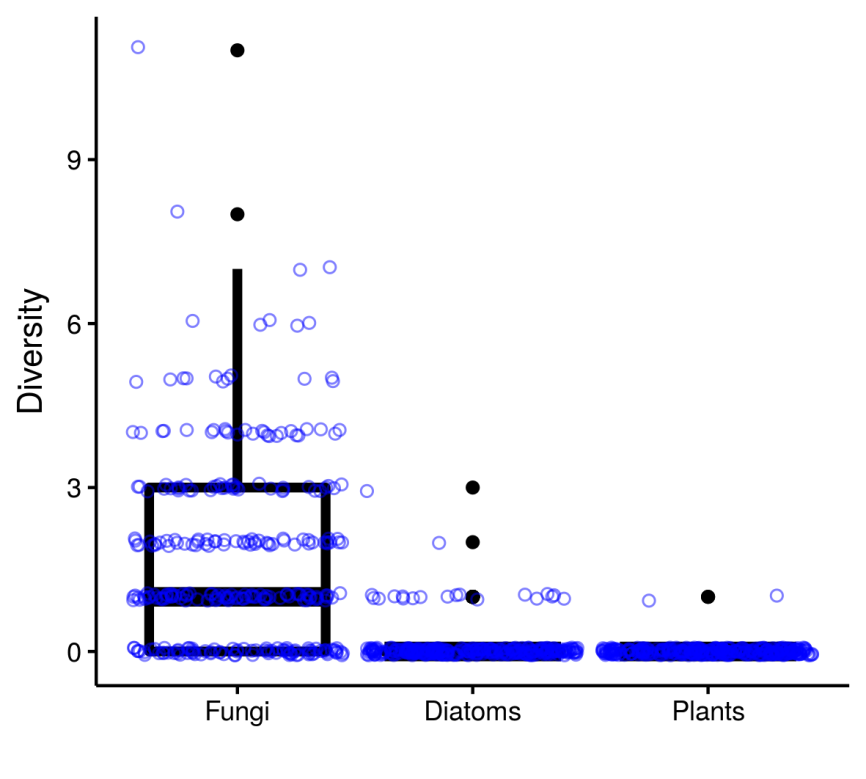
**

**Figure S2.** Barplot depicting the prevalence of food resources found in the microscopic assessment of gut contents of feather mites species inhabiting species of Passeriformes. Error lines represent confidence intervals (95%).


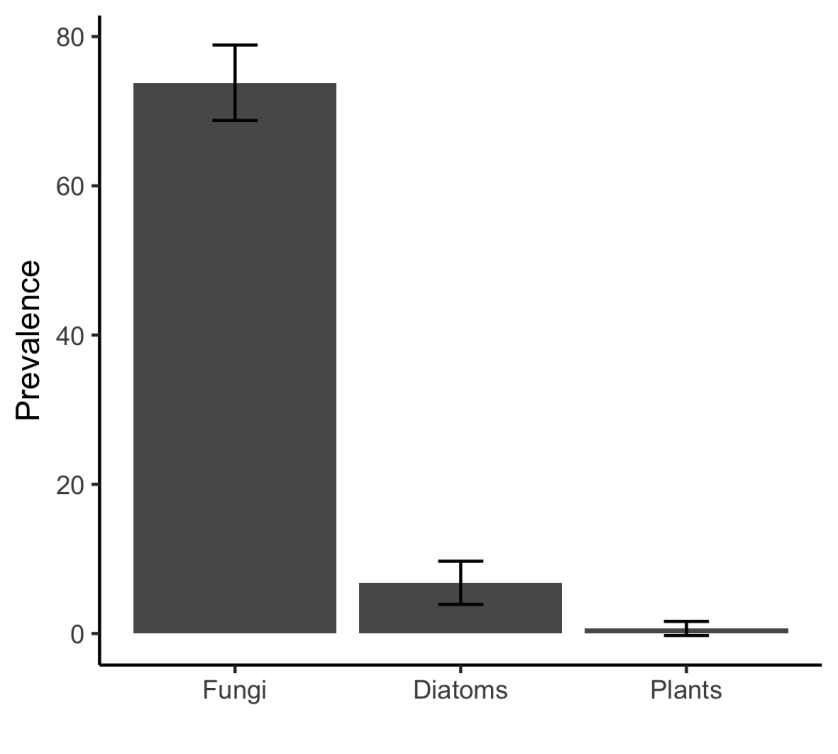


**Figure S3.** Stacked bar plots of the fungal genera retrieved from the listed feather mite species. The top plot “Inside” corresponds to the DNA inside mites (i.e., mite samples), while the bottom plot “Outside” corresponds to the DNA from outside the mites (i.e., environmental samples), see Materials and Methods for further details. Low abundance taxa (< 2 %) and legend were not shown for illustrative purposes.


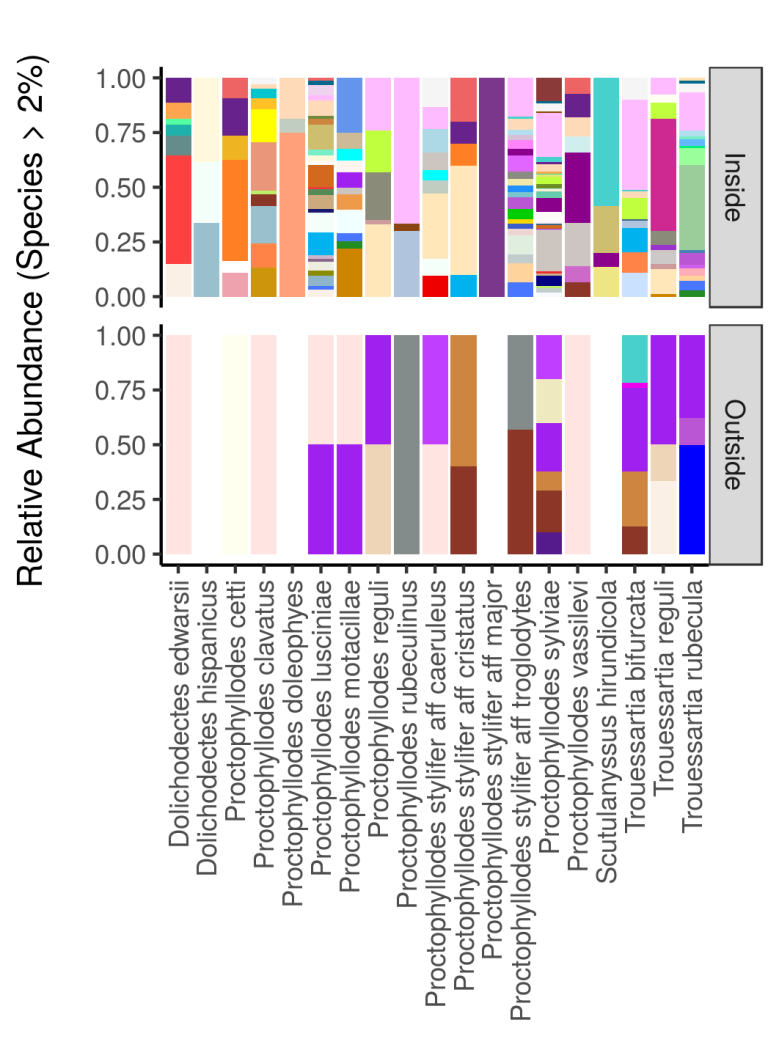


**Figure S4.** Stacked bar plots of the fungal phyla retrieved from the listed feather mite species.


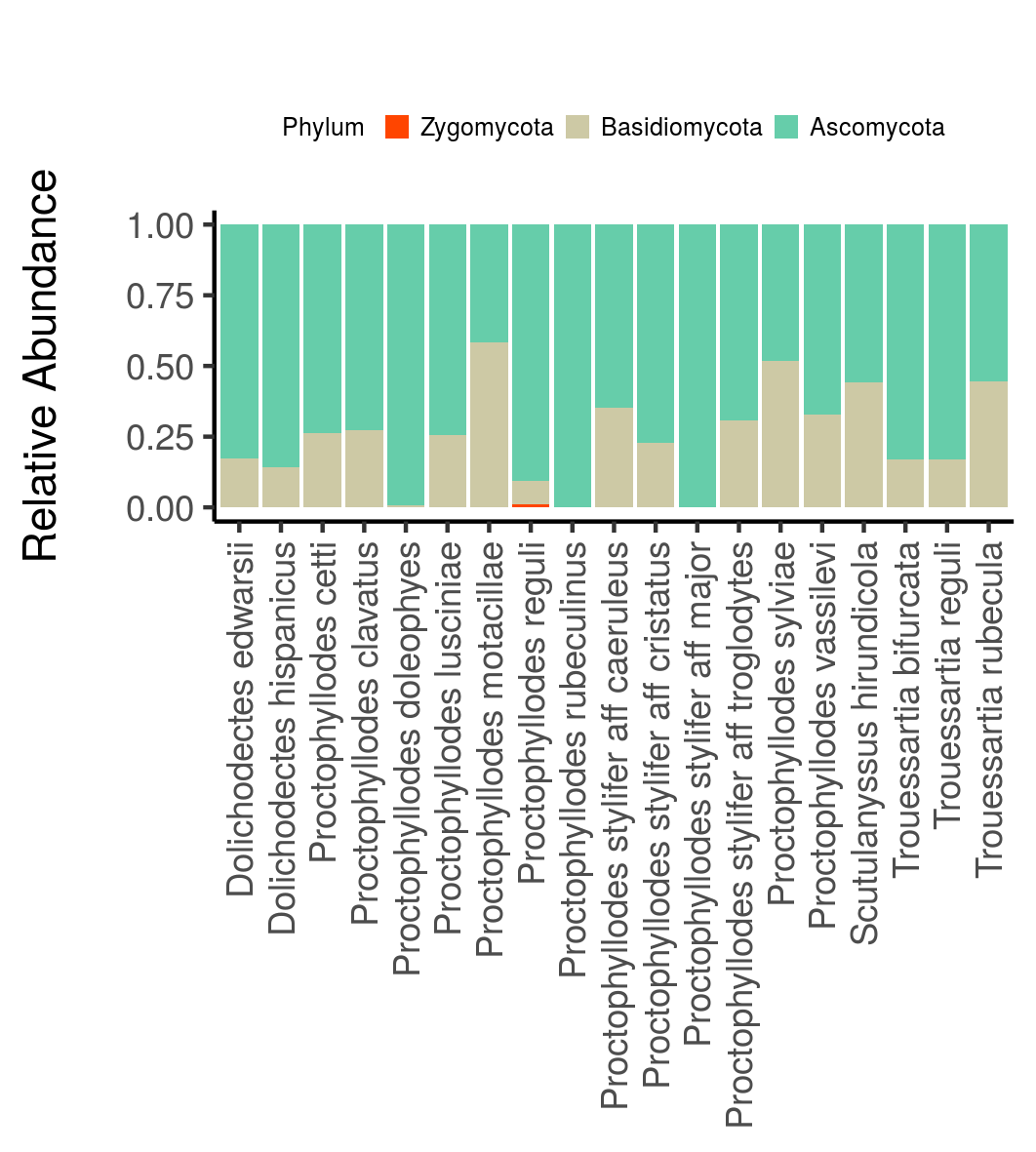


**Figure S5.** Stacked bar plots of the bacterial phyla retrieved from the listed feather mite species.
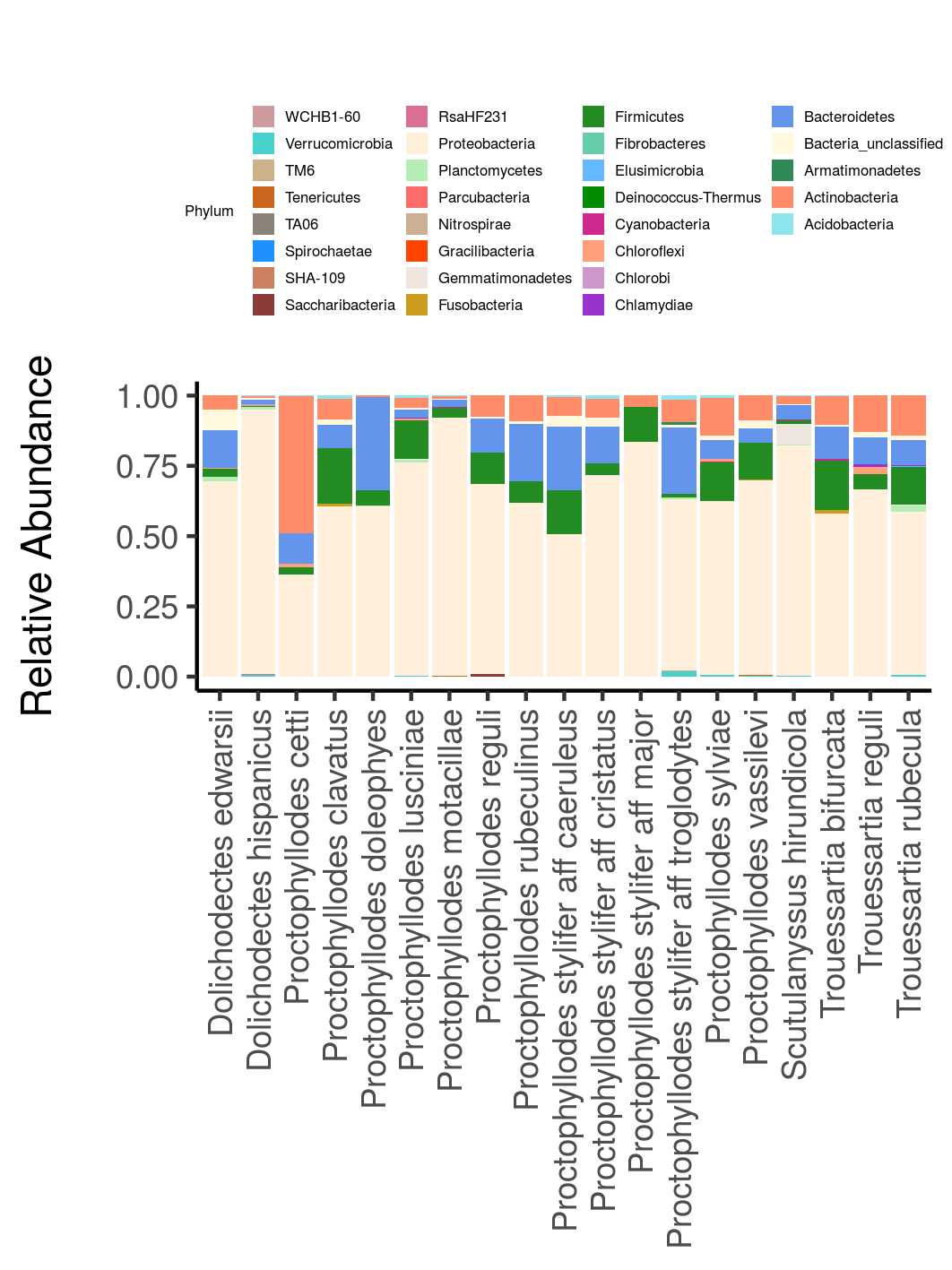


**Figure S6.** Stacked bar plots of the fungal genera retrieved from the listed feather mite infrapopulations. Each row of the plot depicts infrapopulations from the same mite species. Low abundance taxa (< 2 %) were not shown for illustrative purposes.
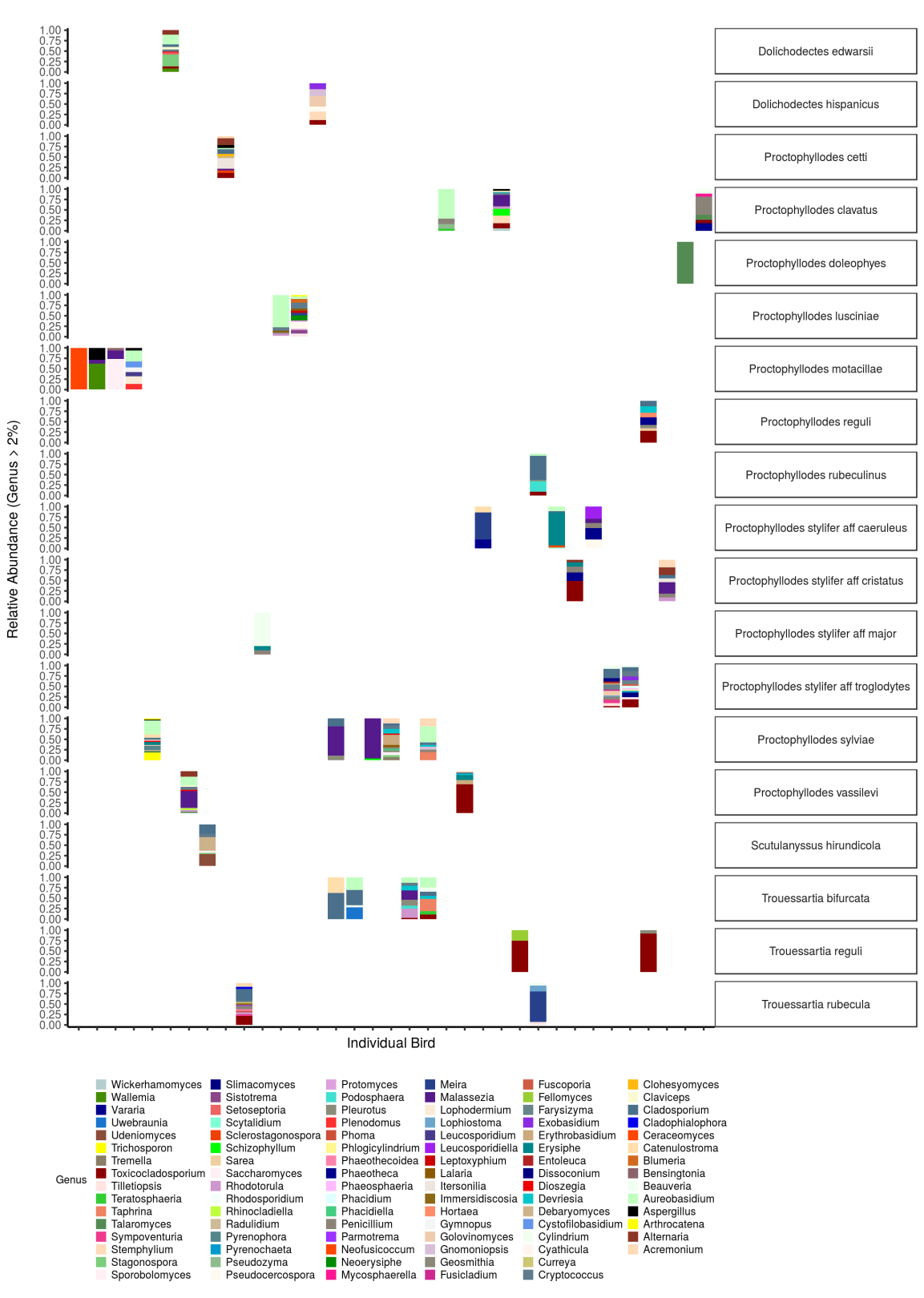


**Figure S7.** Stacked bar plots of the bacterial genera retrieved from the listed feather mite infrapopulations. Each row of the plot depicts infrapopulations from the same mite species. Low abundance taxa (< 2 %) were not shown for illustrative purposes.

**
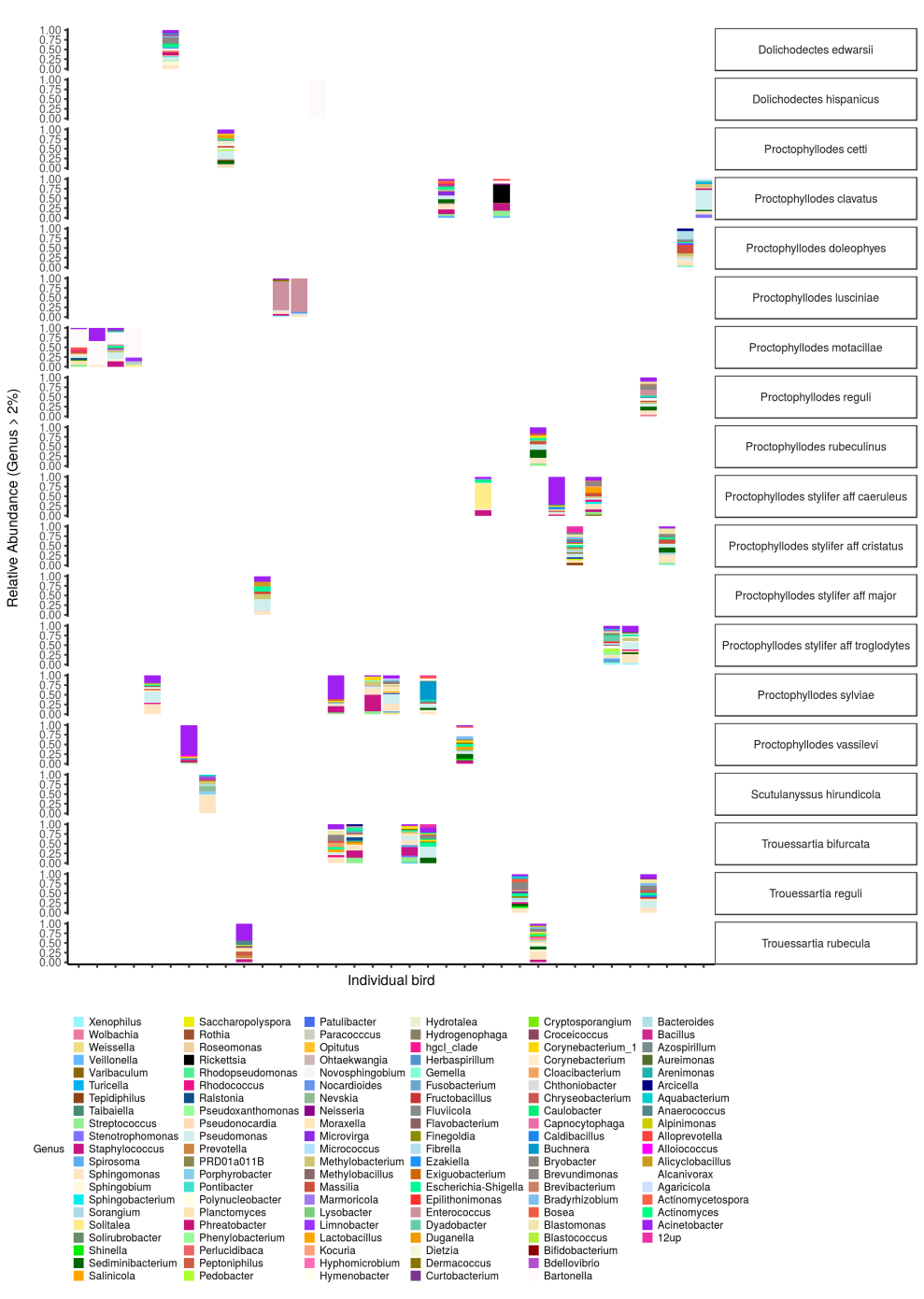
**

**Figure S8.** Stacked bar plots of the bacterial genera retrieved from the listed feather mite infrapopulations excluding those OTUs that appeared at the external sample (See Material and Methods for details). Each row of the plot depicts infrapopulations from the same mite species. Low abundance taxa (< 5 %) were not shown for illustrative purposes.


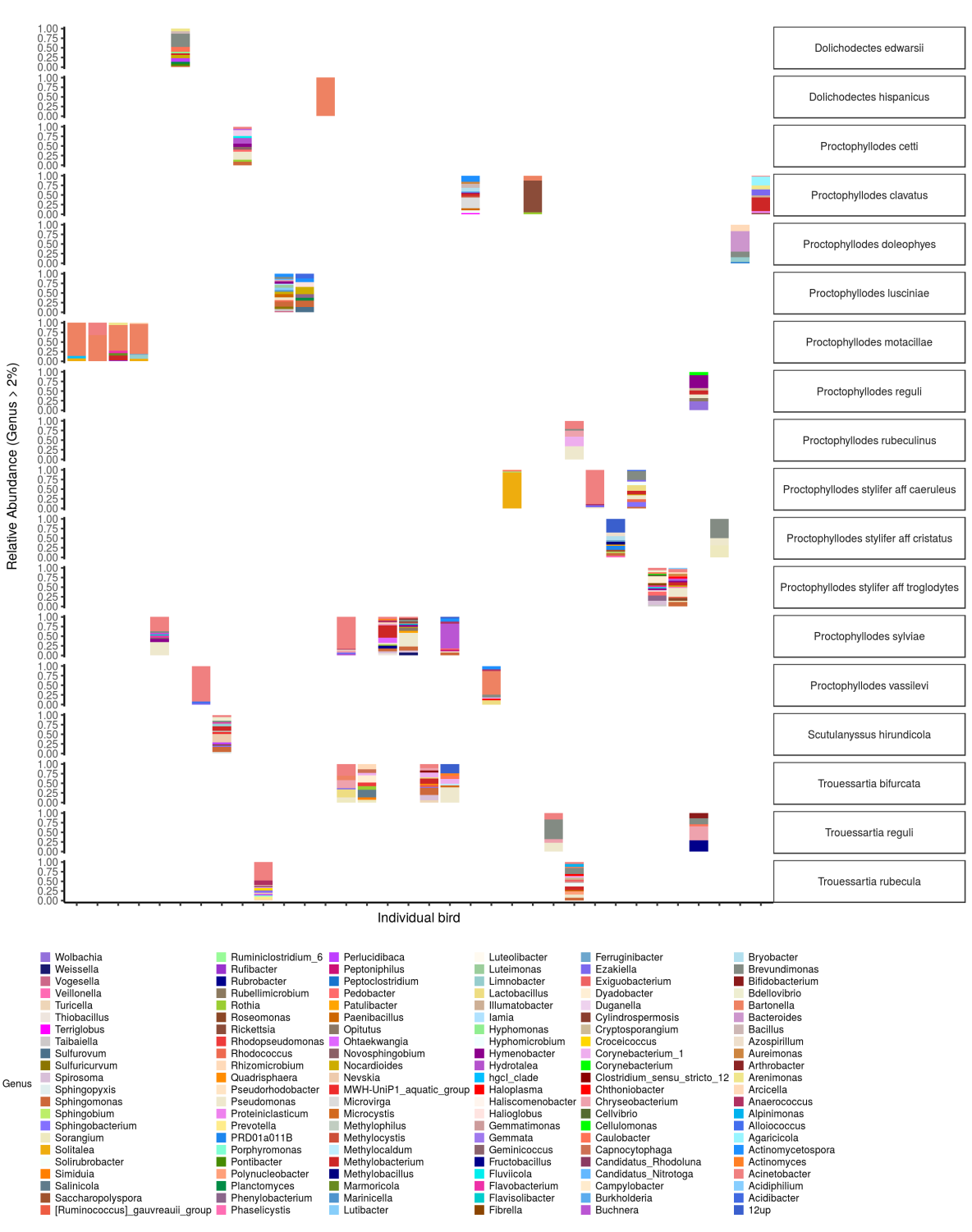


**Figure S9**. Principal coordinates analysis (PCoA) of bacterial communities from the listed feather mite infrapopulations, excluding those OTUs that appeared at the external sample (See Material and Methods for details) : First row, samples colored by mite species and (a) based on Bray and (b) Jaccard distances, respectively; Second row, samples colored by bird species and c) based on Bray and (d) Jaccard distances respectively. OTUs counts were scaled to the smallest library following McMurdie *et al.* 2014 and Denef *et al.* (2016).


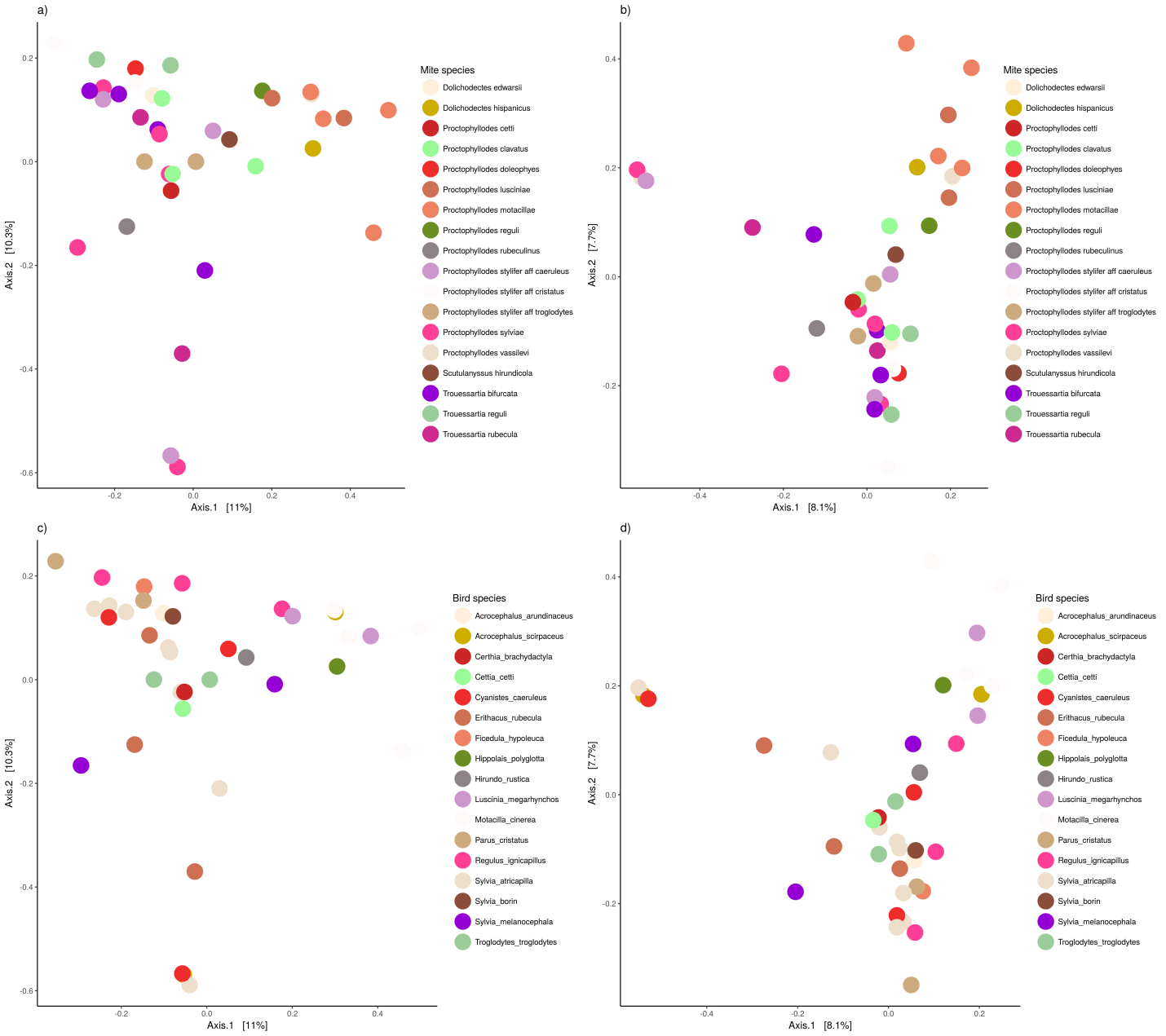


**Figure S10.** Photo showing an example of gut content items categorized as “unidentifiable”.


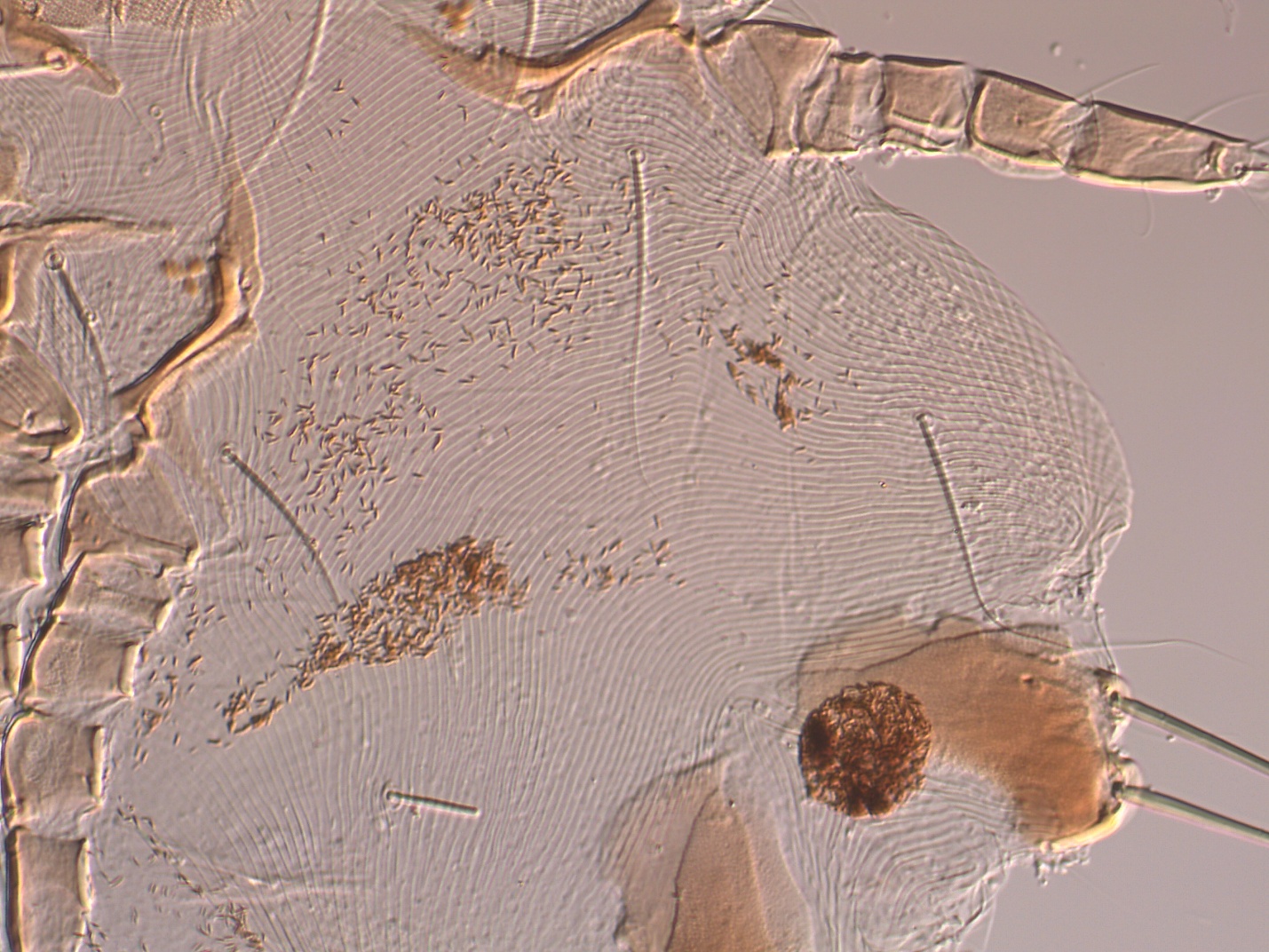


**Figure S11.** Photo showing an example of gut content items categorized as oily glob with inclusions (arrow) (*Proctophyllodes* ex. *Dendroica tigrina*) (HP0941008).


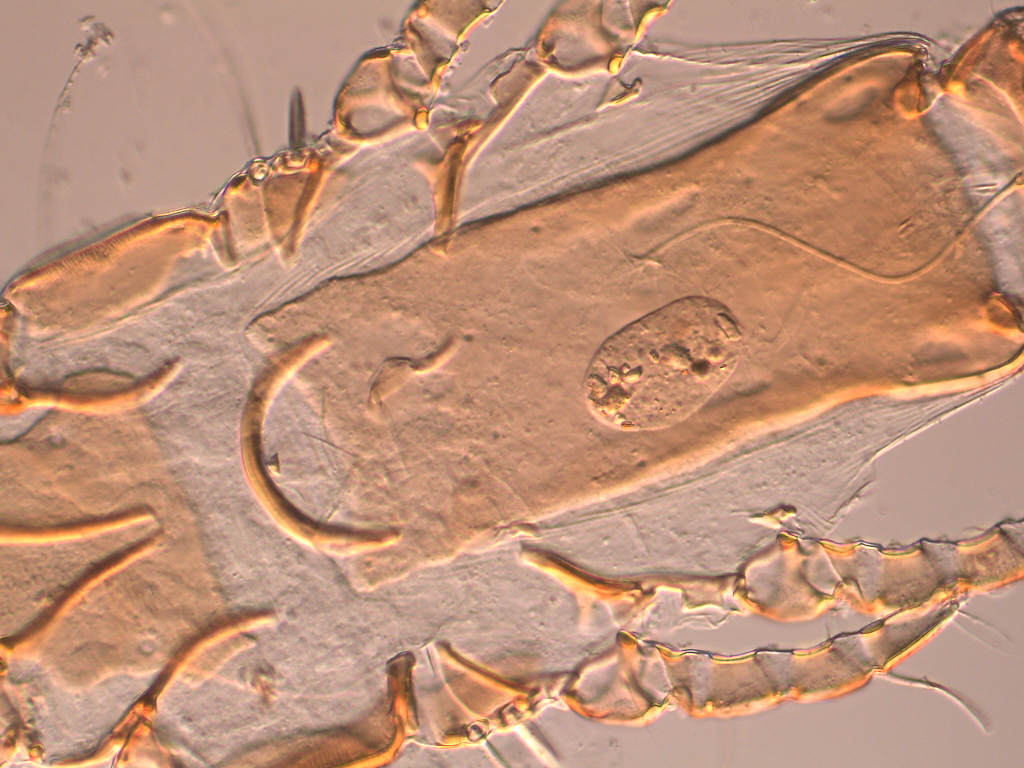

Supplement: Supplementary file 1 [file MEC-28-203-s001.docx]
